# A Co_3_O_4_ Nanoparticle-Modified Screen-Printed Electrode Sensor for the Detection of Nitrate Ions in Aquaponic Systems

**DOI:** 10.3390/s22249730

**Published:** 2022-12-12

**Authors:** Nguyen Thi Dieu Thuy, Xiaochan Wang, Guo Zhao, Tingyu Liang, Zaihan Zou

**Affiliations:** 1 College of Engineering, Nanjing Agricultural University, Nanjing 210031, China; 2 College of Artificial Intelligence, Nanjing Agricultural University, Nanjing 210031, China

**Keywords:** screen-printed electrodes, ion-selective electrode, all-solid-state electrode, nitrate ion detection, cobalt oxide

## Abstract

In this study, a screen-printed electrode (SPE) modified with cobalt oxide nanoparticles (Co_3_O_4_ NPs) was used to create an all-solid-state ion-selective electrode used as a potentiometric ion sensor for determining nitrate ion (NO_3_^−^) concentrations in aquaculture water. The effects of the Co_3_O_4_ NPs on the characterization parameters of the solid-contact nitrate ion-selective electrodes (SC-NO_3_^−^-ISEs) were investigated. The morphology, physical properties and analytical performance of the proposed NO_3_^−^-ion selective membrane (ISM)/Co_3_O_4_ NPs/SPEs were studied by X-ray diffraction (XRD), energy-dispersive spectroscopy (EDS), transmission electron microscopy (TEM), scanning electron microscopy (SEM), electrochemical impedance spectroscopy (EIS), cyclic voltammetry (CV), potentiometric measurements, and potentiometric water layer tests. Once all conditions were optimized, it was confirmed that the screen-printed electrochemical sensor had high potential stability, anti-interference performance, good reproducibility, and no water layer formation between the selective membrane and the working electrode. The developed NO_3_^−^-ISM/Co_3_O_4_ NPs/SPE showed a Nernstian slope of −56.78 mV/decade for NO_3_^−^ detection with a wide range of 10^−7^–10^−2^ M and a quick response time of 5.7 s. The sensors were successfully used to measure NO_3_^−^ concentrations in aquaculture water. Therefore, the electrodes have potential for use in aquaponic nutrient solution applications with precise detection of NO_3_^−^ in a complicated matrix and can easily be used to monitor other ions in aquaculture water.

## 1. Introduction

Aquaponics is a new type of circulating aquaculture technology that was created by combining two agricultural engineering technologies, i.e., aquaculture and hydroponics [1]. The ammonia nitrogen concentration in traditional aquaculture water, which is delivered to the hydroponic agriculture system of the aquaponic system, increases when fish excreta accumulate and gradually increases the toxicity of the aquaculture water. The ammonia nitrogen in water is decomposed by bacteria into nitrite, which is then decomposed into nitrate by nitrifying bacteria. Nitrate can be directly absorbed by plants and utilized as a metabolite [2], but widespread nitrate accumulation in various water resources is now considered one of the biggest threats to aquatic life worldwide. An increasing body of information has been curated to reveal the effects of nitrates on fish growth and health [3], which have shown that fish suffer negative effects in health, behavior, and physiology from nitrate exposure [4,5,6]. Therefore, detecting nitrate in aquaculture solutions is critical for improving the quality of fish health. In recent years, there have been numerous reports and reviews of sophisticated methods for nitrate determination, and researchers have created a number of analytical techniques for detecting nitrate from various sources. These methods include electrochemical detection [7,8], spectrophotometry [9,10], chromatography [11], and capillary electrophoresis [12]. Until now, spectrophotometry has remained the most popular on-site nitrate detection method in aquaculture because of its simplicity, cost-effectiveness, and robustness. However, it has a number of drawbacks, including inaccuracy and susceptibility to interference.

Ion-selective electrodes (ISEs) are generally employed for environmental [13,14] and clinical [15] applications because of their quick manufacturing methods, good sensitivity, high selectivity, low cost, disposability, miniaturization, and ability to suit the needs of smart sensing devices. Traditional ISEs rely on liquid contact electrodes, which results in complex designs, poor mechanical flexibility, difficult maintenance, and large sizes [16]. Scholars have conducted advanced research and produced solid-contact ISEs (SC-ISEs) that, instead of an internal solution, have an ion-to-electron transducer sandwiched between a conducting element and an ion-selective membrane (ISM), which is helpful for overcoming the aforementioned challenges [17]. In an all-solid-state ISE, the potential at the interface between the ISM and the solid electrode should be kept constant to accurately detect the target ion activity in an aqueous sample solution. To achieve this, a solid-state transfer layer that can execute ion–electron transfer to a solid conductive substrate and a polymer ISM has been created [18]. For ion-to-electron transducers, different solid-contact layers of conducting polymers, such as those comprising polythiophene, polyaniline, and polypyrrole, have been used thus far [19]. However, the potential shifts of all-solid-state ISEs are impacted by carbon dioxide, oxygen, and light when utilizing conductive polymers [20]. The use of metallic oxide-based materials as ion-to-electron transducers has become more widespread with the development of nanomaterials [21]. Due to their outstanding physical and chemical properties, they are frequently employed to create sensors with different charge storage capacities [22,23]. Among them, cobalt oxide (Co_3_O_4_) has a high specific capacitance (3560 Fg^−1^), and its electrochemical performance is primarily determined by the structural morphology and electronic state (Co^3+^ or Co^2+^) of the material. Furthermore, Co_3_O_4_ is easy to synthesize, and various synthetic methods, including hydrothermal [24], coprecipitation [25], and sol-gel reactions [26], have been employed to develop various forms of Co_3_O_4_ nanocomposites used to improve sensor performance, lithium-ion batteries, catalytic materials, and supercapacitors [27].

Flexible and disposable screen-printed electrodes (SPEs) have been used for sensor development because they can be mass-produced on a large scale at a low cost [28]. SPEs typically consist of a chemically inert substrate in which three electrodes (a working electrode, reference electrode, and counter electrode) have been screen-printed [29,30]. Many articles on SPEs have described environmental [31], food and beverage [32], heavy metal [33], and biomedical [34] applications. Due to the advantages of SPEs, they are likely to be developed as ISEs to monitor aquaculture conditions.

In this work, Co_3_O_4_ NPs were synthesized by a hydrothermal method. The produced Co_3_O_4_ NPs were characterized by EDS, SEM, TEM, and XRD. Then, using a drop-casting approach, an SPE was modified with the Co_3_O_4_ NPs to fabricate an electrode for the electrochemical detection of NO_3_^−^ions in an aquaculture solution. The fabrication of the NO_3_^−^-ISM/Co_3_O_4_ NPs/SPE was realized by optimizing crucial parameters. Electrochemical impedance spectroscopy (EIS), potentiometry, potentiometric water layer tests, and chronopotentiometry were also conducted to assess the feasibility and reliability of the proposed NO_3_^−^-ISM/Co_3_O_4_ NPs/SPE, which demonstrated good NO_3_^−^ detection performance. Finally, the developed sensors were used to detect NO_3_^−^ in real aquaculture water samples.

## 2. Materials and Methods

### 2.1. Reagents and Instruments

Cobalt (II) acetate tetrahydrate (C_4_H_14_CoO_8_, 99.5%), tetrahydrofuran (THF), ethanol (C_2_H_5_OH, 90%), ammonia (NH_4_OH, 25%), high-molecular-weight polyvinyl chloride (PVC), and other analytical grade chemical reagents were obtained from Sinopharm Chemical Reagent Co., Ltd. (Beijing, China) and used without further purification. Tridodecylmethylammonium nitrate (MTDDA-NO_3_^−^) was purchased from Santa Cruz Biotechnology Co., Ltd. (Santa Cruz, CA, USA). o-Nitrophenyl octyl ether (o-NPOE, 98%) was provided by Shanghai Aladdin Bio-Chem Technology Co., Ltd. (Shanghai, China). Screen-printed electrodes (SPEs) were purchased from Nanjing YunYou Biotechnology Co., Ltd. (Nanjing, China). These electrodes belong to a single-channel three electrode system. The working electrode and counter electrode were made of carbon ink. Silver chloride ink was used as the reference electrode. The aquaponics nutrient solution came from an aquaponics system installed on the roof of the Boyuan Building, College of Engineering, Pukou Campus, Nanjing Agriculture University. Domestic wastewater, tap water, and Yangtze River water samples were collected from the staff area building at Pukou Campus of Nanjing Agricultural University, the city water supply, and the riverbank near Pukou Jiangtan Park in Nanjing City, respectively. Deionized water (18.2 MΩ) was used for all experiments. The X-ray diffraction (XRD) pattern of Co_3_O_4_ was obtained with a Malvern Panalytical XRD instrument (Malvern, Worcestershire, UK). Scanning electron microscopy (SEM) images were obtained with a ZEISS Gemini 300 field-emission scanning electron microscope (ZEISS, Oberkochen, Germany) operated at 3 kV. Energy dispersive spectroscopy (EDS) was conducted using an Oxford Xplore system attached to the FE-SEM instrument. Transmission electron microscopy (TEM) was performed with a JEOL-2100F high-resolution field-emission transmission electron microscope operated at 200 kV. Electrochemical characterizations, such as EIS, CV, potentiometric measurements, and chronopotentiometry, were performed with a CHI660D electrochemical workstation (Shanghai CH Instruments, Shanghai, China).

### 2.2. Synthesis of Cobalt Oxide Nanoparticles

Co_3_O_4_ NPs were synthesized in this study by using a one-step hydrothermal process from the literature [35]. Briefly, 2 g of C_4_H_14_CoO_8_ was dissolved in 100 mL of ethanol (C_2_H_5_OH), and then 10 mL of ammonia (NH_4_OH) solution was added while stirring vigorously. The suspension was transferred to a 250 mL autoclave and stirred for 10 min. The autoclave was sealed and heated at 150 °C for 3 h and then cooled to room temperature. Finally, the product was centrifuged and thoroughly washed several times with ethanol and deionized water, and subsequently dried at 80 °C for 3 h.

### 2.3. Preparation of Nitrate Ion-Selective Electrodes

**Figure 1:**
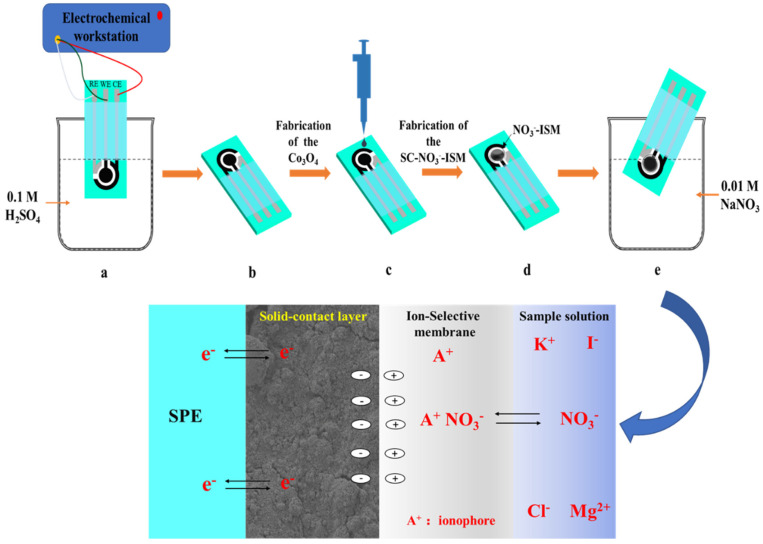
Schematic overview of fabrication of the NO_3_^−^-ISM/Co_3_O_4_ NPs/SPEs.

The process for preparing the nitrate ISEs can be described as follows: the SPEs were activated in 0.1 M H_2_SO_4_ by cyclic voltammetry before detection (a) and then left to dry for a few minutes (b). Three milligrams of Co_3_O_4_ nanoparticles were ultrasonically dispersed in 1 mL of deionized water for 30 min to create a homogeneous dispersion. In total, three 3 µL aliquots of the Co_3_O_4_ NP dispersion were drop-cast onto the SPE (c). Finally, two 5 µL aliquots of a solution containing 6% MTDDA-NO_3_^−^, 65% o-NOPE, and 29% PVC in 1.5 mL tetrahydrofuran (THF) [36] were drop-cast on top of the Co_3_O_4_ NPs and left to dry overnight and form the two NO_3_^−^-ISM layers (d). All of the prepared NO_3_^−^-ISM/Co_3_O_4_ NPs/SPEs were conditioned in 10^−2^ M NaNO_3_ for 24 h before use (e). The NO_3_^−^-ISM/Co_3_O_4_ NPs/SPEs were fabricated as depicted in Figure 1.

## 3. Results and Discussions

### 3.1. Parameter Optimization for NO_3_^−^-ISM/Co_3_O_4_ NPs/SPE Fabrication

**Figure 2:**
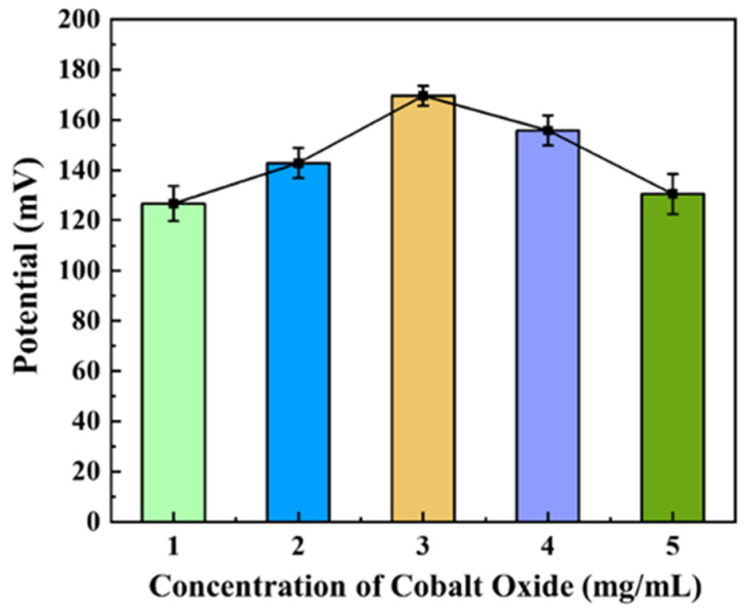
Potentials at various Co_3_O_4_ concentrations.

In this study, the Co_3_O_4_ concentration was optimized to improve the detection performance of the proposed NO_3_^−^-ISM/Co_3_O_4_ NPs/SPE, as shown in Figure 2. The optimization range of the Co_3_O_4_ concentration was from 1 mg/mL to 5 mg/mL. For extremely low concentrations of Co_3_O_4_, the amount of Co_3_O_4_ remaining after drying was very low. Additionally, Co_3_O_4_ with a similar dispersion grade could not be completely concentrated on the electrode surface. However, when the Co_3_O_4_ concentration was too high, the Co_3_O_4_ formed a thick layer on the electrode surface and reduced the electrode sensitivity. As such, the electrochemical properties resulting from Co_3_O_4_ modification in these two scenarios were not good enough and high levels of noise and low sensitivity resulted. Finally, we selected 3 mg/mL as the optimal Co_3_O_4_ concentration.

### 3.2. Physical Properties of the Synthesized Co_3_O_4_ Nanoparticles

**Figure 3:**
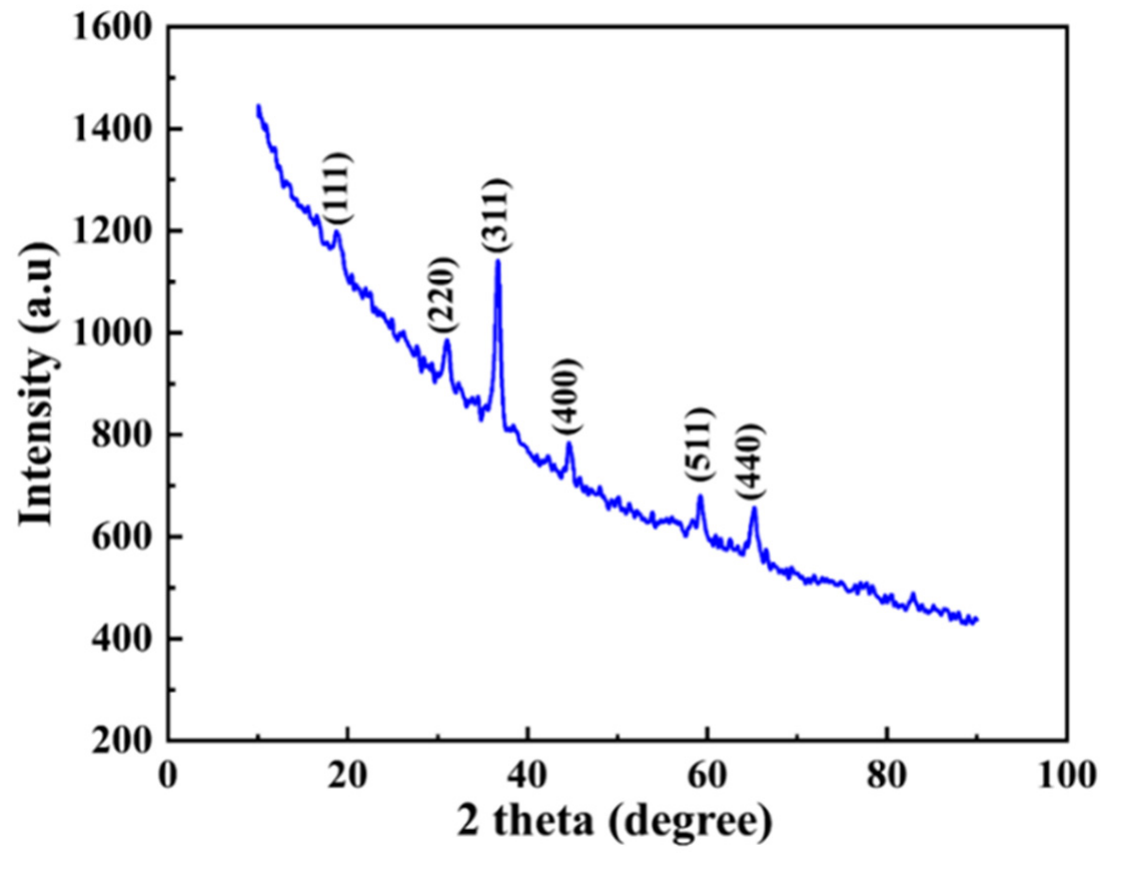
X-ray diffraction (XRD) pattern for Co_3_O_4_ NPs.

Figure 3 shows that the XRD pattern showed peaks at 2θ = 18.72°, 30.98°, 36.73°, 44.41°, 58.96°, and 64.96°, which can be assigned to the (111), (220), (311), (400), (511), and (440) crystal planes of Co_3_O_4_ (JCPDS card No. 42-1467) [24]. There were no peaks visible for additional impurity phases, demonstrating that the product was extremely pure. These results showed that Co_3_O_4_ was effectively synthesized and possessed a pure spinel structure.

**Figure 4:**
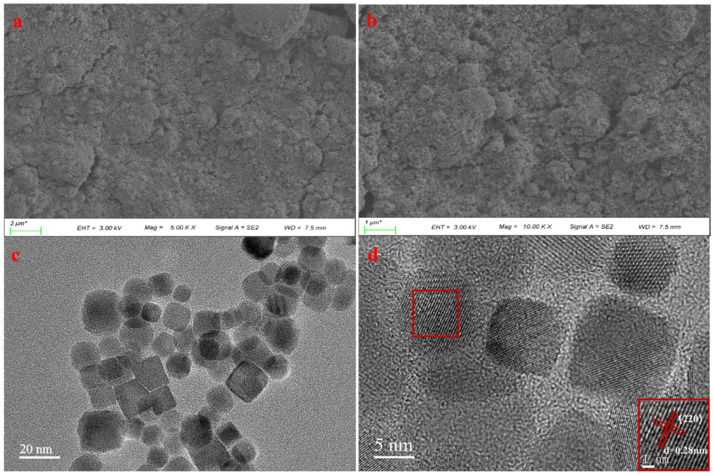
(**a**,**b**) SEM images of the Co_3_O_4_ NPs with different magnifications. (**c**,**d**) TEM images of the Co_3_O_4_ NPs showing the interplanar spacing (220 plane).

SEM images of the Co_3_O_4_ NPs are displayed in Figure 4a,b. The SEM results reveal the Co_3_O_4_ NP surface morphology, which was mostly homogenous and aggregative in nature after dispersion. This showed that the bulk state of the as-prepared Co_3_O_4_ NPs was separate [37]. This morphology is advantageous for the fabrication of electrodes for supercapacitor applications; in general, a supercapacitor requires a material with a large surface area [38]. However, SEM cannot be used to determine whether the Co_3_O_4_ existed as nanoparticles. The physical properties of the Co_3_O_4_ were determined using TEM analysis, as shown in Figure 4c,d. According to the TEM analysis, the synthesized nanoparticles were uniform, with an average particle size of approximately 12 nm. Figure 4d depicts the plane spacing of the nanoparticles and the presence of crystals with various orientations. Additionally, the distance between the fringes of two nearby crystals was measured. The measured planar distance of 0.28 nm was assigned to plane 220. These results were consistent with those previously reported [39,40]. The TEM results showed that this preparation method successfully produced small Co_3_O_4_ NPs.

**Figure 5:**
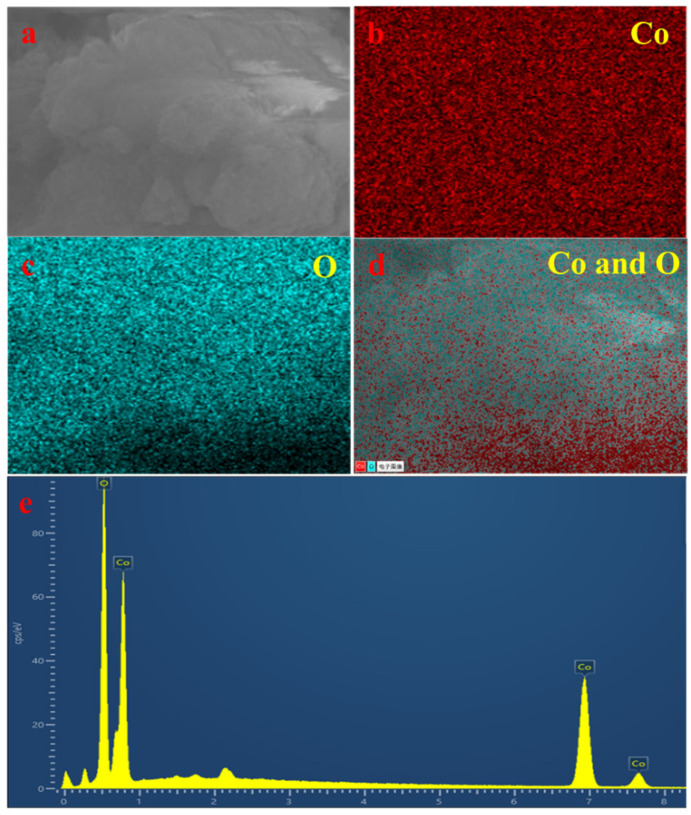
(**a**) SEM image of the selected area for Co_3_O_4_. (**b**,**c**), and (**d**) show the distributions of Co, O, and both atoms within the selected area, respectively. (**e**) EDS spectrum of Co_3_O_4_.

Figure 5 shows the EDS results for Co_3_O_4_. Figure 5b,c, and d show images for a selected area of the Co_3_O_4_ NPs (Figure 5a). In Figure 5e, the elemental composition of the Co_3_O_4_ NPs is clearly shown. This demonstrated that only cobalt and oxygen components were present in the manufactured specimen with no other differentiated components, indicating that the synthesized Co_3_O_4_ NPs were extremely clean [25].

### 3.3. Electrochemical Analysis of the Proposed Sensors

**Figure 6:**
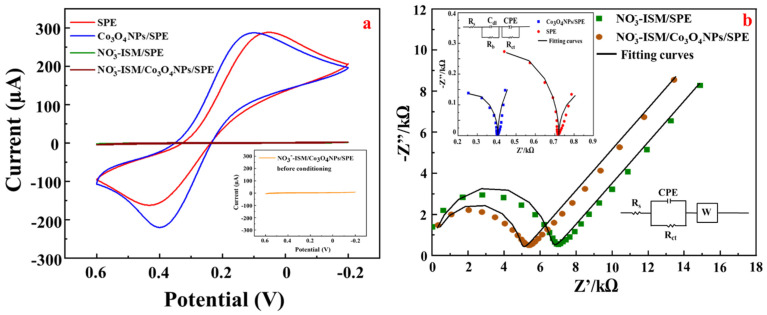
(**a**) CV curves for SPE (red line), Co_3_O_4_ NPs/SPE (blue line), NO_3_^−^-ISM/SPE (green line), NO_3_^−^-ISM/Co_3_O_4_ NPs/SPE with conditioning (brown line), and NO_3_^−^-ISM/Co_3_O_4_ NPs/SPE without conditioning (inset). (**b**) Nyquist plots for SPE (red points), Co_3_O_4_ NPs/SPE (blue points), NO_3_^−^-ISM/SPE (green points), and NO_3_^−^-ISM/Co_3_O_4_ NPs/SPE (brown points) and their corresponding electrochemical impedance spectra fitting curves (black line).

Cyclic voltammetry (CV) was performed in a solution of 5 mM K_3_[Fe(CN)6]^3−/4−^ containing 0.1 M KCl. In each case, three cycles were performed over the potential range −0.1–0.6 V at a scan rate of 0.05 V s^−1^. Figure 6a shows the different CVs of the SPE and Co_3_O_4_ NPs/SPE. After modification, the redox current of the Co_3_O_4_ NPs/SPE was 288.8 µA (blue line), higher than that of the bare SPE at 285.2 µA (red line). The good dispersion of Co_3_O_4_ ensured adhesion to the electrode surface with good stability. Therefore, Co_3_O_4_ NPs also have the advantage of stabilizing the electron transfer rate on the SPE surface. After modifying the nitrate ISM, we carried out two experiments in which we performed CV measurements before and after electrode conditioning. The inset in Figure 6a shows the CV curve obtained before conditioning. The redox current of the NO_3_^−^-ISM/Co_3_O_4_ NPs/SPE was found to be 0.417 µA (before conditioning), which was the same as that of the electrode after conditioning at 0.625 µA. The results for both cases showed that the CV current density was very low when the membrane was modified. This was due to the ion-selective membrane’s nonconductive properties.

EIS measurements were performed over frequencies ranging from 10 Hz to 10^6^ Hz with an open-circuit voltage of 0.358 V and an excitation amplitude of 0.005 V. Figure 6b depicts the equivalent circuit used to fit the resulting Nyquist plots, in which R_b_, R_ct_, R_s_, C_dl_, CPE, and W represent the bulk membrane resistance together with the contact resistance between the underlying conductor and the ISM, the charge-transfer resistance, the solution resistance, the double-layer capacitance, the constant phase angle element, and the Warburg impedance, respectively [41]. The R_ct_ values of the SPE (red points) were approximately 0.72 kΩ. For the Co_3_O_4_ NPs/SPE (blue points), the semicircle diameter was increased at high frequencies compared to that for the SPE, which corresponded to an R_ct_ of 0.4 kΩ. Because the ISM was nonconductive, the impedance increased after the addition of the nitrate ISM. The R_ct_ values of the NO_3_^−^-ISM/SPE (green points) were 7.01 kΩ and were reduced to approximately 5.41 kΩ for the NO_3_^−^-ISM/Co_3_O_4_ NPs/SPE (brown points). These results showed that the presence of a Co_3_O_4_ NP transduction layer effectively reduced the high-frequency resistance of the solid-state nitrate ISE because the presence of an ion–electron transduction layer in the Co_3_O_4_ NPs improved the charge-transfer rate between the solid conductive substrate and the polymer nitrate ISM. Additionally, compared to the NO_3_^−^-ISM/SPE, the NO_3_^−^-ISM/Co_3_O_4_ NPs/SPE had a larger electric double-layer capacitance and a lower charge-transfer resistance between the SPE and ISM interface. Furthermore, the spectrum curve of NO_3_^−^-ISM/Co_3_O_4_ NPs/SPE was closer to the actual axis of the impedance spectrum than that of NO_3_^−^-ISM/SPE in the low-frequency region, showing that NO_3_^−^-ISM/Co_3_O_4_ NPs/SPE had a higher low-frequency electric double-layer capacitance.

### 3.4. Chronopotentiometric Test

**Figure 7:**
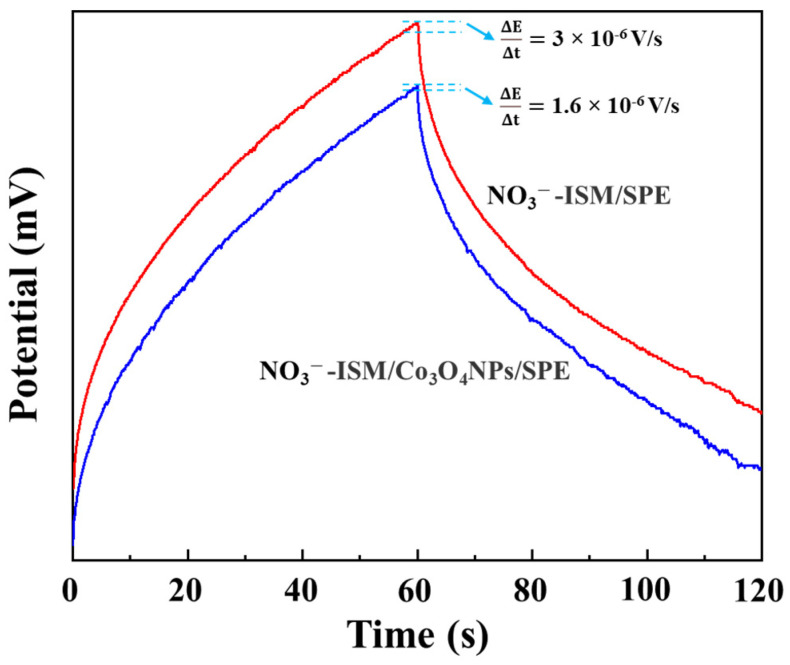
Chronopotentiograms for the NO_3_^−^-ISM/SPE (red) and NO_3_^−^-ISM/Co_3_O_4_ NPs/SPE (blue) recorded in 0.01 M NaNO_3_ solution by applying alternating currents of ±1 nA for 60 s.

To estimate the effect of the solid-contact layer of the Co_3_O_4_ NPs on the capacitance of the NO_3_^−^-ISE and investigate the stability of the prepared NO_3_^−^-ISM/Co_3_O_4_ NPs/SPE, chronopotentiometry with a constant current was used to investigate and record the potential change of the prepared NO_3_^−^-ISM/Co_3_O_4_ NPs/SPE. In the chronopotentiometric spectrum, the potential change rate can be described as ΔE/Δt = I/C (where ΔE is the potential change, Δt is the time variation, I is the applied current, and C is the low-frequency capacitance), and it can be used to determine electrode stability and is a significant index [42]. Chronopotentiometry experiments were performed by recording the potentials of the ISE in the current range of ±1 nA in a 0.01 M NaNO_3_ solution—the experimental results are shown in Figure 7. In the chronopotentiometric spectrum, the difference between the two types of electrodes occurred mostly due to their low-frequency capacitance differences. The SPE potential change rate reached 3 × 10^−6^ V/s when there was no Co_3_O_4_ transducing layer, while a smaller potential change rate of 1.6 × 10^−6^ V/s was recorded for the Co_3_O_4_ NPs/SPE. Finally, the capacitance of the NO_3_^−^-ISM/Co_3_O_4_ NPs/SPE was calculated to be 62.5 µF, which was higher than that of the NO_3_^−^-ISM/SPE, which was 33 µF. It can be seen from the above results that the nitrate ISE modified with Co_3_O_4_ NPs showed a significantly higher capacitance and lower potential change rate than the SPE under the same developed conditions. These results confirmed that the Co_3_O_4_ NPs were successfully used as a transducer material.

### 3.5. Potentiometric Water Layer Tests

The water layer that forms at the membrane–inner electrode interface during the working process of the solid-state ISE will obviously affect the potential stability of the SC-ISE. Therefore, potentiometric water layer test experiments are an important component of investigating the performance of a solid-state ISE. For the potentiometric water layer test experiment, we carried out the test according to the literature method [43]. If there is a water layer, a positive potential drift will occur when the electrode is moved from the main ion solution to the interference ion solution. When the electrode is removed from the interference ion solution and placed back in the main ion solution, a negative potential drift will occur. In this experiment, NO_3_^−^-ISM/Co_3_O_4_ NPs/SPE and NO_3_^−^-ISM/SPE were activated in 0.1 M NaNO_3_ solution for 12 h before the water layer tests.

**Figure 8:**
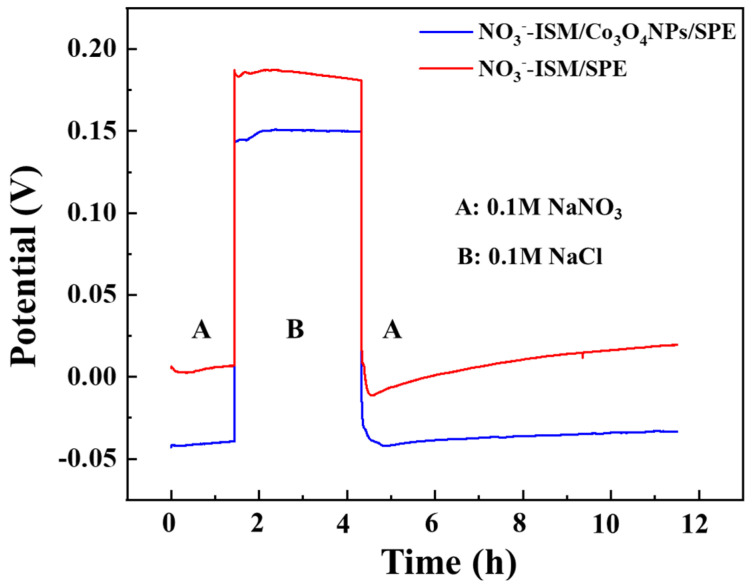
Potentiometric water layer tests of NO_3_^−^-ISM/Co_3_O_4_ NPs/SPE (blue) and NO_3_^−^-ISM/SPE (red). The measurements were completed by switching the electrodes between 0.1 M NaNO_3_ and 0.1 M NaCl solutions.

The specific experimental process was as follows, using NO_3_^−^-ISM/Co_3_O_4_ NPs/SPE as an example: First, the activated NO_3_^−^-ISM/Co_3_O_4_ NPs/SPE was immersed in a 0.1 M NaNO_3_ solution. After 1.5 h, the NO_3_^−^-ISM/Co_3_O_4_ NPs/SPE was moved from the 0.1 M NaNO_3_ solution to a 0.1 M NaCl solution. After that, the NO_3_^−^-ISM/Co_3_O_4_ NPs/SPE was transferred from the 0.1 M NaCl solution back to the 0.1 M NaNO_3_ solution for approximately 7 h. We recorded the potential change curves for the NO_3_^−^-ISM/Co_3_O_4_ NPs/SPE over time throughout the process. The water layer test results are shown in Figure 8. At t = 1.5 h, the NaNO_3_ solution was replaced by the NaCl solution, and NO_3_^−^-ISM/Co_3_O_4_ NPs/SPE and NO_3_^−^-ISM/SPE displayed rapid potential drops, demonstrating that the prepared ISEs have good selectivity for nitrate ions. In addition, the potential response of NO_3_^−^-ISM/SPE exhibited a positive drift, while the potential response of NO_3_^−^-ISM/Co_3_O_4_ NPs/SPE changed only slightly during this process. At t = 4 h, when the NO_3_^−^-ISM/Co_3_O_4_ NPs/SPE was removed from the NaCl solution and placed in the NaNO_3_ solution, its potential returned to the initial value, and the potential drift was not obvious in subsequent tests. These results revealed that the potential drift of the NO_3_^−^-ISM/SPE was greater. The tests indicated that using Co_3_O_4_ NPs as the solid transduction layer can effectively reduce the existence of the water layer between the solid conductive substrate and the ISM.

### 3.6. Selectivity of the NO_3_^−^-ISM/SPE and NO_3_^−^-ISM/Co_3_O_4_ NPs/SPE

Selectivity, as the name indicates, is another important index that characterizes these sensors. The selectivities of the NO_3_^−^-ISM/SPE and the NO_3_^−^-ISM/Co_3_O_4_ NPs/SPE were estimated with the selectivity coefficient values obtained in relation to ion interference. The separable solution method was used to determine the selectivity coefficients of the tested electrodes [44], and their potentiometric selectivity coefficients were calculated using the Nicolskii–Eisenman equation:

(1) logKI,Jpot=EJ−EI·ZI·F2.303·R·T+logaJaIZIZJ

where I is the primary ion, J is the interfering ion, E is the potential, T is the temperature, F is Faraday’s constant, R is the ideal gas constant, z is the valency of the ion, and a is the ion activity. Table 1 displays the results of the experiment, which confirmed that the studied ISEs showed high selectivity for their primary ion, and even the addition of the Co_3_O_4_ NPs did not impact their selectivity.

### 3.7. Effect of pH on the Response of the NO_3_^−^-ISM/Co_3_O_4_ NPs/SPE for NO_3_^−^ Detection

**Figure 9:**
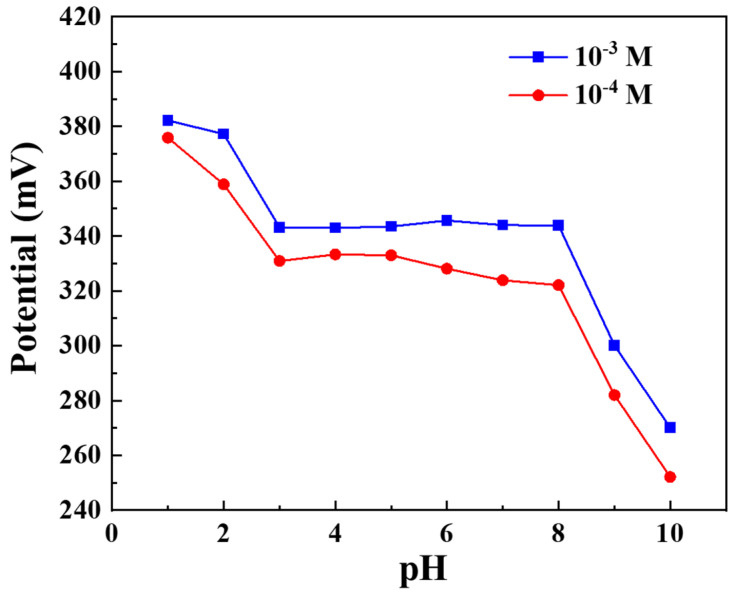
Effect of pH on the NO_3_^−^-ISM/Co_3_O_4_ NPs/SPE potential response.

The most crucial element influencing electrode detection is the pH value. In this work, nitrate concentrations varying between 10^−4^ and 10^−3^ M were used to examine the impact of the pH on the electrode potential. The pH was changed from 1.0 to 10 by adding small amounts of HNO_3_ and NaOH. The effect of pH on the electrode potential response is shown in Figure 9. The findings showed that the electrode potential was relatively stable between pH 3.0 and pH 8.0. Potential drift occurred when the pH value was too high or too low, which can be caused by hydrogen–ion interference.

### 3.8. Enhanced Sensing Principle of the NO_3_^−^-ISM/Co_3_O_4_ NPs/SPE

**Figure 10:**
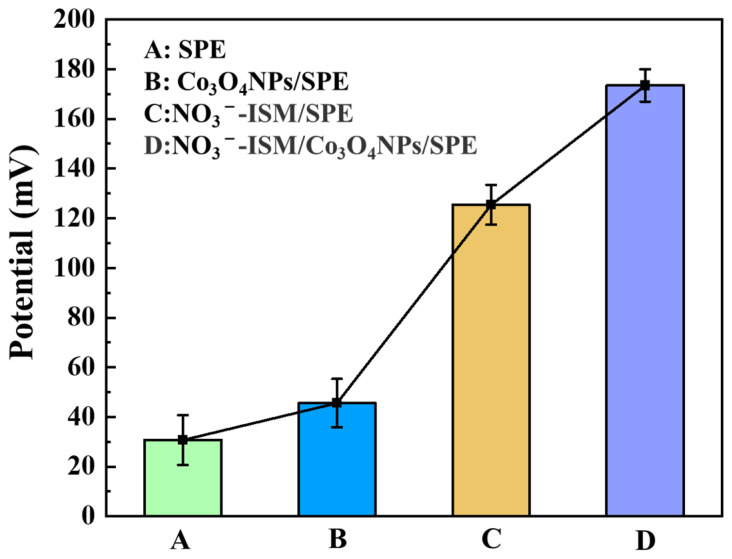
Potential response comparison of the SPE, Co_3_O_4_ NPs/SPE, NO_3_^−^-ISM/SPE and NO_3_^−^-ISM/Co_3_O_4_ NPs/SPE.

Figure 10 shows a comparison of the potential responses of the SPE, Co_3_O_4_ NPs/SPE, NO_3_^−^-ISM/SPE, and NO_3_^−^-ISM/Co_3_O_4_ NPs/SPE. The average potential response data for six different concentrations of NO_3_^−^, 10^−7^, 10^−6^, 10^−5^, 10^−4^, 10^−3^, and 10^−2^ M, were investigated. The electrodes without the nitrate ISM modification, i.e., the SPE and Co_3_O_4_ NPs/SPE, showed almost no response to nitrate ions, which gave an extremely low potential response (Figure 10A,B), while the NO_3_^−^-ISM modification significantly increased the sensitivities of the electrodes (Figure 10C,D). Therefore, the NO_3_^−^-ISM used in this study showed good sensitivity for detecting nitrate ions. Additionally, the NO_3_^−^-ISM/Co_3_O_4_ NPs/SPE exhibited a greater potential response than the NO_3_^−^-ISM/SPE, which indicated that Co_3_O_4_ was an excellent ion and electron transport material because it has a cubic spinel crystal structure and is a magnetic p-type semiconductor oxide [45]. Furthermore, Co_3_O_4_ has intriguing electrochemical, electronic, optical, catalytic, and electrocatalytic properties that could be used to increase electrolyte diffusion and provide more ion transport paths in supercapacitors.

### 3.9. Potentiometric Characteristics

**Figure 11:**
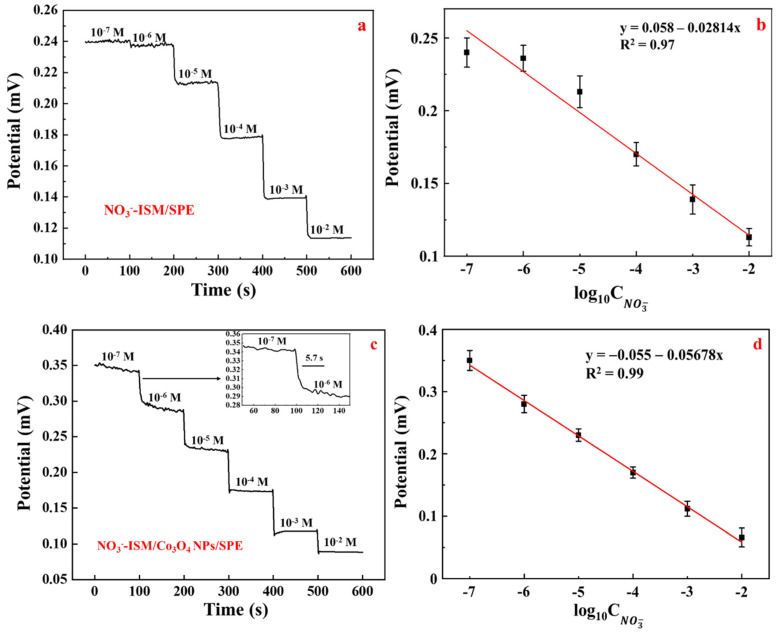
Dynamic potentiometric response and calibration curve for the NO_3_^−^-ISM/SPE (**a**,**b**) and the NO_3_^−^-ISM/Co_3_O_4_ NPs/SPE (**c**,**d**).

In this study, the responses of the NO_3_^−^-ISM/SPE and the new SC-NO_3_^−^-ISE to nitrate ions were confirmed by using a series of NaNO_3_ solutions with concentrations ranging from 10^−2^ to 10^−7^ M. The potential responses of the NO_3_^−^-ISM/SPE and SC-NO_3_^−^-ISE transduced by the Co_3_O_4_ NPs are shown in Figure 11. Figure 11a,c shows the dynamic potential responses of the NO_3_^−^-ISM/SPE and NO_3_^−^-ISM/Co_3_O_4_ NPs/SPE. The results revealed that the NO_3_^−^-ISM/SPE response was very small at low concentrations. Compared with the NO_3_^−^-ISM/SPE, the NO_3_^−^-ISM/Co_3_O_4_ NPs/SPE had a shorter response time and achieved potential balance in various nitrate ion solutions even at low concentrations. Moreover, the NO_3_^−^-ISM/Co_3_O_4_ NPs/SPE showed a steady potential response to nitrate ions as the nitrate ion concentration was changed. The inset shows the potential response over time when the concentration was changed by one order of magnitude (10^−7^ to 10^−6^ M), which is considered to be the electrode’s slowest response in the linear range. After changing the concentration by an order of magnitude, the time required to reach a steady-state signal was typically less than 60 s. These results showed that the NO_3_^−^-ISM/Co_3_O_4_ NPs/SPE had a very short response time, and a stable signal was obtained in approximately 5.7 s, which is sufficient for many applications. The calibration curves for the two electrodes are shown in Figure 11b,d. The NO_3_^−^-ISM/SPE showed a Nernstian response of −28.14 mV/decade (R^2^ = 0.97) with a detection limit of 2.69 × 10^−5.6^ M and a quantification limit of 8.17 × 10^−5.6^ M. The NO_3_^−^-ISM/Co_3_O_4_ NPs/SPE showed a Nernstian response slope of −56.78 mV/decade (R^2^ = 0.99) with a detection limit of 1.04 × 10^−8^ M and a quantification limit of 3.18 × 10^−8^ M, which are better than those of the NO_3_^−^-ISM/SPE. These findings demonstrate that the electrode response was significantly improved by using CO_3_O_4_ as a solid-contact layer.

**Figure 12:**
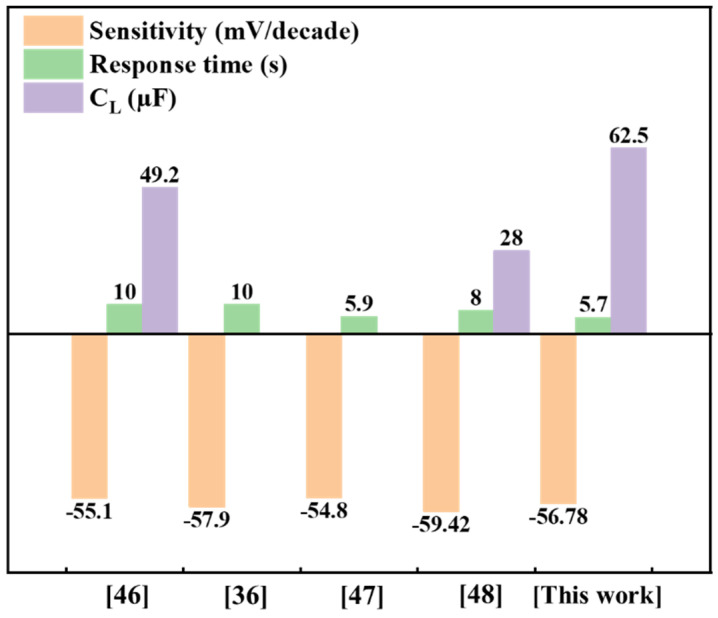
Performance comparison of different SC-NO_3_^−^-ISEs. Refs. [36,46,47,48] are compared with this research.

Figure 12 shows a comparison of different solid-state nitrate ISEs that have recently been reported in the literature for NO_3_^−^ detection [36,46,47,48]. The results show that the proposed NO_3_^−^-ISM/Co_3_O_4_ NPs/SPE has superior sensitivity, response time, and high capacitance compared to the other electrodes, which demonstrates that the proposed sensors have great sensitivity, rapid response, marginally improved capacitance, and improved selectivity for nitrate ions.

### 3.10. Lifetime Study for the NO_3_^−^-ISM/Co_3_O_4_ NPs/SPE

**Figure 13:**
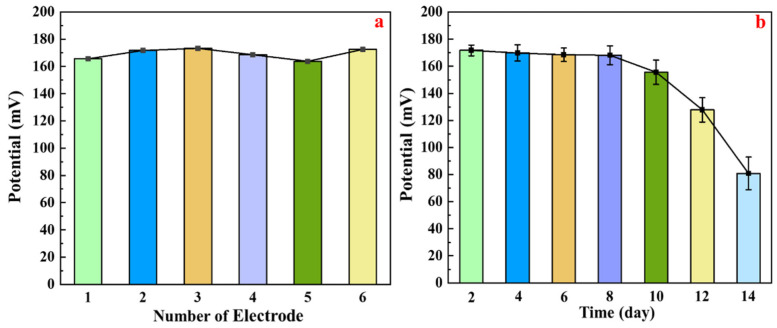
(**a**) Responses of six different NO_3_^−^-ISM/Co_3_O_4_ NPs/SPEs and (**b**) responses of the NO_3_^−^-ISM/Co_3_O_4_ NPs/SPE over fourteen days.

An investigation was conducted to evaluate the lifetime of the proposed NO_3_^−^-ISM/Co_3_O_4_ NPs/SPE. The electrodes were calibrated in NaNO_3_ solutions with concentrations ranging from 10^−2^ to 10^−7^ M after conditioning in 10^−2^ M NaNO_3_ for 24 h, and the results are shown in Figure 13a. All six electrodes showed similar responses, with a standard deviation of 1.39. Moreover, the measurements were carried out every two days while using the same electrode to investigate the stability of the proposed NO_3_^−^-ISM/Co_3_O_4_ NPs/SPE, as shown in Figure 13b. Based on an analysis of the recorded data, the NO_3_^−^-ISM/Co_3_O_4_ NPs/SPE had good stability over the time range 0–8 d. However, there was an obvious decrease in the sensor response, which reached approximately 50% after 8 d. These results indicated that the proposed NO_3_^−^-ISM/Co_3_O_4_ NPs/SPE has an active life of approximately 8 d.

### 3.11. Analyses of Real Water Samples from an Aquaponic System

To investigate the utility of the proposed NO_3_^−^-ISM/Co_3_O_4_ NPs/SPE for monitoring NO_3_^−^ levels in aquaponic water, two real water samples obtained from an aquaponic system were used. Simultaneously, we examined other water samples to confirm the electrode’s functionality, such as domestic wastewater, tap water, and Yangtze River water samples. In addition, the detection results obtained with the NO_3_^−^-ISM/Co_3_O_4_ NPs/SPE were compared with those from a traditional detection method, i.e., spectrophotometry. Table 2 displays the composition of the aquaculture water used in this work.

As shown in Table 3, the results for determinations of nitrate ions in the aquaponic system with the solid-state NO_3_^−^-ISM/Co_3_O_4_ NPs/SPE were essentially consistent with the data obtained using spectrophotometry, demonstrating that our sensor can be utilized to efficiently detect NO_3_^−^ in aquaculture water.

## 4. Conclusions

In this work, SPEs were used to develop new NO_3_^−^-ISM/Co_3_O_4_ NPs/SPEs for the detection of NO_3_^−^ in aquaponics water, and Co_3_O_4_ NPs were used to modify the electrode surfaces to inhibit formation of an aqueous layer in the membrane–inner electrode interface. The electrode capacitance was increased by orders of magnitude through the introduction of Co_3_O_4_ NPs, which was required for stable output. Our sensors also exhibited an excellent sensitivity of −56.78 mV/decade and a quick response time of 5.7 s, and XRD, EDS, SEM, and TEM were used to confirm the structural integrity. The key parameters for fabrication of the NO_3_^−^-ISM/Co_3_O_4_ NPs/SPE were optimized to improve the NO_3_^−^ detection performance, which combined high potential stability, strong reproducibility, and low interference. The outcomes for the analyses of real water samples from an aquaponic system demonstrated that the NO_3_^−^-ISM/Co_3_O_4_ NPs/SPE developed in this paper has good commercial potential for the detection of nitrate ions in aquaponic water.

## Figures and Tables

**Table 1:** Selectivities of the NO_3_^−^-ISM/SPE and NO_3_^−^-ISM/Co_3_O_4_ NPs/SPE.

Interfering Ion	Concentration (mg L^−1^)	Selectivity Coefficient	
		NO_3_^−^-ISM/SPE	NO_3_^−^-ISM/Co_3_O_4_ NPs/SPE
SO_4_^2−^	0.1	−5.7 ± 0.2	−5.4 ± 0.3
Br^−^	0.1	−3.5 ± 0.2	−3.8 ± 0.2
Cl^−^	0.1	−3.0 ± 0.3	−3.1 ± 0.1
CO_3_^2−^	0.1	−4.3 ± 0.2	−4.8 ± 0.2
I^−^	0.1	−3.1 ± 0.1	−2.8 ± 0.1
K^+^	0.1	−5.8 ± 0.1	−5.7 ± 0.2
Na^+^	0.1	−4.9 ± 0.2	−4.5 ± 0.2
Mg^+^	0.1	−4.1 ± 0.3	−3.6 ± 0.3

**Table 2:** Aquaculture water composition.

Aquaculture Water Composition Parameters	Concentration	Value
NO_3_^−^	mg/L	5.27
NO_2_^−^	mg/L	0.11
NH_4_^+^	mg/L	21.2
pH		7.06
SS	mg/L	16
BOD	mg/L	9.1
DO	mg/L	0.66
Chlorophyll a	µg/L	1.85

**Table 3:** Results from determinations of NO_3_^−^ in an aquaponic system.

Sample	NO_3_^−^-ISM/Co_3_O_4_ NPs/SPE (×10^−5^ M)	Spectrophotometer (×10^−5^ M)
Aquaculture water	7.98 ± 0.06	8.50 ± 0.02
Yangtze River water	1.81 ± 0.04	1.69 ± 0.03
Domestic water	1.58 ± 0.02	1.62 ± 0.02
Tap water	2.02 ± 0.02	1.97 ± 0.01

(Means ± standard deviations, n = 3).

## Data Availability

Not applicable.
